# 1266. Melatonin for Renal Protection of Patients Treated with Polymyxin B: A Double Blind Randomized Clinical Trial

**DOI:** 10.1093/ofid/ofab466.1458

**Published:** 2021-12-04

**Authors:** Maria Helena Rigatto, Pedro Bergo, Giulia Baldissera, Eduarda Beck, Leonardo David, Lucas Santoro, Diego Rodrigues Falci, Wolnei Caumo, Alexandre Zavascki

**Affiliations:** 1 Hospital São Lucas da PUCRS, Porto Alegre, Rio Grande do Sul, Brazil; 2 PUCRS, Porto Alegre, Rio Grande do Sul, Brazil; 3 Hospital de Clinicas de Porto Alegre, Porto Alegre, Rio Grande do Sul, Brazil; 4 Hospital de Clínicas de Porto Alegre, Porto Alegre, Rio Grande do Sul, Brazil

## Abstract

**Background:**

Polymyxins are one of the last resort treatments for carbapenem resistant gram-negative infections. Nephrotoxicity is its main adverse effect and has been related to oxidative stress mechanisms. Melatonin was associated to reduction in polymyxins nephrotoxicity in animal studies. Our objective is to evaluate the effect of melatonin on renal protection of patients receiving polymyxin B.

**Methods:**

We did a single center, double blind, randomized clinical trial (NCT03725267) of melatonin 30mg versus placebo for patients treated with polymyxin B from October 2018 to April 2021, in Porto Alegre, Brazil. Patients ≥18 years old, receiving polymyxin B for ≤48 hours, who accepted informed consent terms were included and excluded if intensive care unit (ICU) admission at enrollment, estimated glomerular rate estimated glomerular rate < 10ml/min, dialysis or previous melatonin use. Treatment with melatonin or placebo was randomized in blocks of 4 and maintained until the end of polymyxin B treatment of for a maximum of 14 days. Our main outcome was any level of nephrotoxicity by RIFLE score. Secondary outcomes were renal failure and need for dialysis. We estimated a sample size of 100 patients, however the study had to be stopped earlier due to recruitment restrictions imposed by the COVID-19 pandemic.

**Results:**

Eighty-eight patients were randomized, 44 received melatonin and 44 received identical placebo pills. Patients had a mean age of 63.6±17.3 years, 60.2% were male, and had a median Charlson index of 5 (3-8.3). Most infections (79.5%) were microbiologically confirmed, having 68.6% Klebsiella sp isolated. Urinary tract accounted for47.7% of infection sites. Median time of polymyxin B therapy was 9.1±6.6 days. Combination therapy was prescribed for 89.8% of patients and 38.6% received at least another nephrotoxic drug. All variables were equally distributed among groups. Nephrotoxicity rates occurred in 23 of 44 (52.3%) in both groups, P=0.99. Patients who developed renal failure were 8(18.2%) vs 9(20.5%) and dialysis occurred in 4(9.1%) vs 5 (11.4%) of melatonin and placebo groups respectively.

**Conclusion:**

Melatonin did not show a clinically significant renal protective effect in patients treated with polymyxin B.

**Disclosures:**

**Maria Helena Rigatto, MD, PhD**, **CNPq** (Grant/Research Support) **Diego Rodrigues Falci, MD, MSc, PhD**, **Gilead Sciences** (Grant/Research Support, Scientific Research Study Investigator, Speaker’s Bureau)**GSK** (Grant/Research Support, Scientific Research Study Investigator, Advisor or Review Panel member)**MSD** (Speaker’s Bureau)**Pfizer** (Speaker’s Bureau)**United Medical** (Speaker’s Bureau, Other Financial or Material Support) **Alexandre Zavascki, MD, PhD**, **Pfizer** (Grant/Research Support)

